# How does atmospheric pressure cold helium plasma affect the biomechanical behaviour on alkali-lesioned corneas?

**DOI:** 10.1186/s12917-024-03980-6

**Published:** 2024-04-24

**Authors:** Simona Neri, Maria Vittoria Mascolini, Antonella Peruffo, Silvia Todros, Matteo Zuin, Luigi Cordaro, Emilio Martines, Barbara Contiero, Emanuele Luigi Carniel, Ilaria Iacopetti, Marco Patruno, Chiara Giulia Fontanella, Anna Perazzi

**Affiliations:** 1 Department of Animal Medicine, Production and Health, University of Padua, Padova, Italy; 2 Department of Industrial Engineering, University of Padua, Padova, Italy; 3 Centre for Mechanics of Biological Materials, University of Padua, Padova, Italy; 4 Department of Comparative Biomedicine and Food Science, University of Padua, Padova, Italy; 5 RFX (CNR, ENEA, INFN), Padova, Italy; 6 CNR, Institute for Plasma Science and Technology, Padova, Italy; 7 Department of Physics “G. Occhialini”, University of Milano – Bicocca, Milano, Italy

**Keywords:** Porcine cornea, Plasma treatment, Cross-linking, Biomechanics, Ex vivo animal model

## Abstract

**Background:**

Melting corneal ulcers are a serious condition that affects a great number of animals and people around the world and it is characterised by a progressive weakening of the tissue leading to possible severe ophthalmic complications, such as visual impairment or blindness. This disease is routinely treated with medical therapy and keratoplasty, and recently also with alternative regenerative therapies, such as cross-linking, amniotic membrane transplant, and laser. Plasma medicine is another recent example of regenerative treatment that showed promising results in reducing the microbial load of corneal tissue together with maintaining its cellular vitality. Since the effect of helium plasma application on corneal mechanical viscoelasticity has not yet been investigated, the aim of this study is first to evaluate it on ex vivo porcine corneas for different exposition times and then to compare the results with previous data on cross-linking treatment.

**Results:**

94 ex vivo porcine corneas divided into 16 populations (healthy or injured, fresh or cultured and treated or not with plasma or cross-linking) were analysed. For each population, a biomechanical analysis was performed by uniaxial stress-relaxation tests, and a statistical analysis was carried out considering the characteristic mechanical parameters. In terms of equilibrium normalised stress, no statistically significant difference resulted when the healthy corneas were compared with lesioned plasma-treated ones, independently of treatment time, contrary to what was obtained about the cross-linking treated corneas which exhibited more intense relaxation phenomena.

**Conclusions:**

In this study, the influence of the Helium plasma treatment was observed on the viscoelasticity of porcine corneas ex vivo, by restoring in lesioned tissue a degree of relaxation similar to the one of the native tissue, even after only 2 min of application. Therefore, the obtained results suggest that plasma treatment is a promising new regenerative ophthalmic therapy for melting corneal ulcers, laying the groundwork for further studies to correlate the mechanical findings with corneal histology and ultrastructural anatomy after plasma treatment.

## Background

Corneal ulcers affect a great number of people and animals worldwide every year and can lead to severe complications such as visual impairment or blindness when not properly treated [1]. In particular, treating melting infectious keratitis, a condition that implies an imbalance between proteinases and proteinases inhibitors, is becoming increasingly harder. This disease can develop very quickly, even in 24–48 h, triggered and carried on by many types of bacteria, fungi and viruses, such as Pseudomonas spp., Staphylococcus spp., Candida spp., Acanthamoeba spp. and Herpes spp [1]. These microorganisms, together with host activated leukocytes, produce many proteolytic enzymes that can damage and dissolve the corneal stroma, potentially leading to its perforation if not properly regulated [2]. The difficulty in coping with this condition is due to the frightening growth of antibiotic resistance, the increased need of cornea donors, and the rise in the costs of medical and surgical therapies. So, great efforts in regenerative medicine research focus on developing reliable and affordable alternatives to keratoplasty, such as corneal cross-linking (CXL) [3–13], stem cells transplant [14–18], platelet-rich plasma [19, 20], argon laser [21]. Another emerging regenerative treatment is plasma medicine [22]. Plasma medicine makes use of an ionised gas (plasma) directly on biological tissues for therapeutic purposes. Helium, argon, oxygen, nitrogen, or a mixture can be used. The gas flow is ionised passing through an electric field, which induces the gas breakdown and the formation of a low temperature plasma (here the temperature is that of the neutral gas, which remains the dominant component), generating many reactive oxygen and nitrogen species (ROS and RNS). The spreading of these species directly on the surface of damaged tissue improves the healing process by inactivating microorganisms and stimulating cells to produce proinflammatory cytokines and neoangiogenic factors [23–25]. Plasma medicine has been investigated in human skin wound healing in experimental trials on volunteers [23] and in ex vivo human corneas [26–28]. Martines et al. [27] studied the efficacy and safety of a 2 min exposure of human ex vivo corneas to helium cold plasma flow. They showed a ROS-induced antimicrobial effect of the treatment, without any DNA damage in keratocytes and conjunctival fibroblasts and without modifications on corneal cells viability, demonstrating that the use of plasma could be an effective tool for infectious keratitis management. As the main problem of melting corneal ulcers is the softening of the tissue and the modifications in its mechanical functionality that can lead to eyes perforation, the goal of medical research should be the design of a method that strengthens the structure of the corneal stroma. So, it is important to investigate the corneal biomechanical properties in physiological conditions, their changes in tissue strengthening due to melting lesions and different treatments.

Biomechanical tests can be performed on ex vivo porcine corneas that have been already frequently used as a model to estimate the eyes properties of humans and other animals [29–31], despite some differences in thickness between species. Uniaxial tensile tests and inflation tests are the most used methods for ex vivo studies [29, 31–35]. The literature reports few studies on this aspect and with contrasting outcomes [23, 35–37]. Biomechanical evaluation of corneal tissue properties after helium cold plasma exposition is missing in the literature, while it has been investigated for other treatments, such as cross-linking by Fontanella et al. [38]. The above-mentioned paper assessed the biomechanical properties of the corneal tissue of ex vivo healthy *versus* alkali-induced lesioned porcine corneas treated/not treated with CXL, showing a surprising weakening of the lesioned populations despite the treatment [38], a result that is in contrast with the literature.

Indeed, corneal cross-linking has been demonstrated to be effective for the treatment of human and animal collagenasic corneal ulcers, because it seems to increase the stiffness and the resistance of the cornea. This effect is due to a photochemical reaction occurring when the corneal stroma is soaked with riboflavin (Vit. B2) and then radiated with UVA. This interaction generates free oxygen radicals with antimicrobial properties and develops new covalent bonds in the collagen fibrils of the stroma, reinforcing the tissue and influencing its biomechanical functions [29, 31–36]. CXL was originally designed for human keratoconus and then, due to its antimicrobial effects, was applied to infectious keratitis [3–8], also in veterinary ophthalmology [9–13].

So, considering the potential use of plasma medicine for ophthalmic purposes as an alternative to traditional therapies, this study has a twofold purpose. The first aim is to evaluate how the exposition to the ROS produced in a helium plasma modifies the biomechanical properties of the corneal tissue by considering two treatment times and the same experimental conditions and mechanical protocol as the study on CXL [38] The second and collateral aim is to compare the results with those published on CXL treatment [38], in order to assess if there is any difference in corneal tissue relaxation after the two types of processing.

## Results

### Porcine organ-culture preparation procedure

A total number of 52 corneal samples were successfully set up, found clear and without corneal scarring, opacities or injuries. The plasma treatment was correctly performed in all of the samples. All the corneas were stored in the liquid medium for preservation and did not develop any oedema or infection over the period of cultures (7 days). The same condition was observed in the healthy, lesioned and CXL-treated corneal population, as already described in Fontanella et al. [38] thus allowing to join all data for the statistical analysis and the comparison of mechanical behaviour. The populations setting is described in Table 1.

**Table 1:** The table explicits the abbreviations used to identify the population setting

Population	Label
Healthy, Fresh, Not Treated	HFN
Healthy, Fresh, 2 min Plasma Treated	HFP2
Healthy, Fresh, 4 min Plasma Treated	HFP4
Healthy, Fresh, CXL Treated	HFY
Healthy, Cultured, Not Treated	HCN
Healthy, Cultured, 2 min Plasma Treated	HCP2
Healthy, Cultured, 4 min Plasma Treated	HCP4
Healthy, Cultured , CXL Treated	HCY
Lesioned, Fresh, Not Treated	LFN
Lesioned, Fresh, 2 min Plasma Treated	LFP2
Lesioned,Fresh, 4 min Plasma Treated	LFP4
Lesioned, Fresh, CXL Treated	LFY
Lesioned, Cultured, Not Treated	LCN
Lesioned, Cultured, 2 min Plasma Treated	LCP2
Lesioned, Cultured, 4 min Plasma Treated	LCP4
Lesioned, Cultured, CXL Treated	LCY

### Mechanical testing results

#### Stress-strain behaviour

**Fig. 1:**
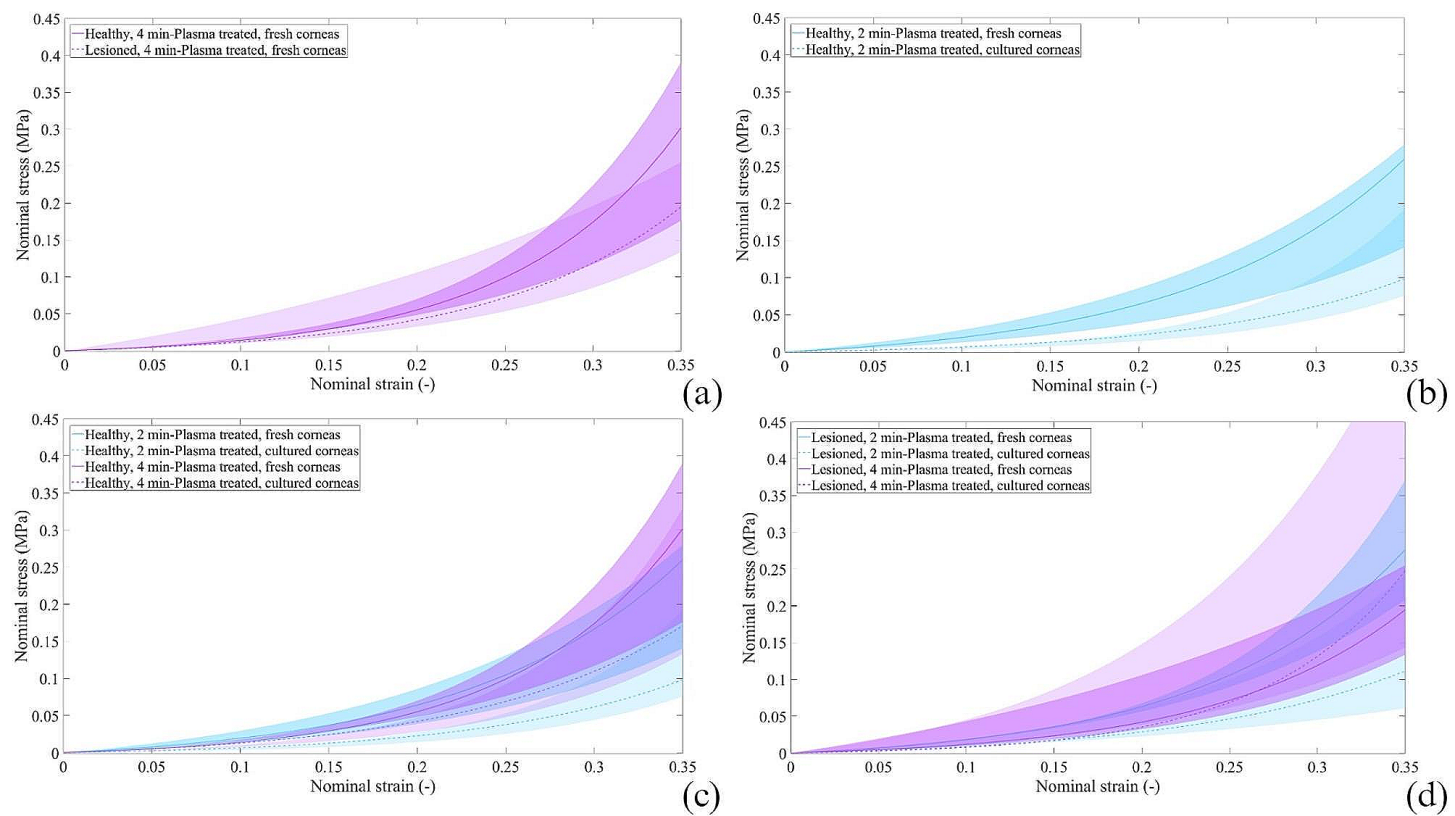
Stress-strain curves at equilibrium resulted for plasma-treated corneas in case of (**a**) induction of lesion, (**b**) culture preservation and different exposition times for (**c**) healthy corneas and (**d**) lesioned corneas. Median curves are reported together with 50% probability scatter bands

The equilibrium stress-strain curves of each group were compared. The median curves for each population are represented by coloured lines and the surrounding coloured area represents the dispersion of the data group (Fig. 1). All the curves show a stiffening trend, so a non-linear behaviour of the corneal tissue. The slope of the curve depends on the tissue stiffness: the greater the steepness of the curve, the higher the tissue stiffness. The induction of a lesion resulted in a lower stiffness if compared to the healthy group, under the same other experimental conditions (Fig. 1a). Moreover, the preservation of corneas in a culture medium resulted in a weakening of the tissue if compared to fresh corneas on equal other conditions (Fig. 1b). The in-culture groups displayed lower values compared to their counterpart fresh ones, except for the LCP4 group which showed higher values (unexpected result). In terms of the exposure time to the plasma treatment (Fig. 1c), the HFP2 group showed lower values of stiffness compared to the HFP4.

#### Viscoelastic behaviour

**Fig. 2:**
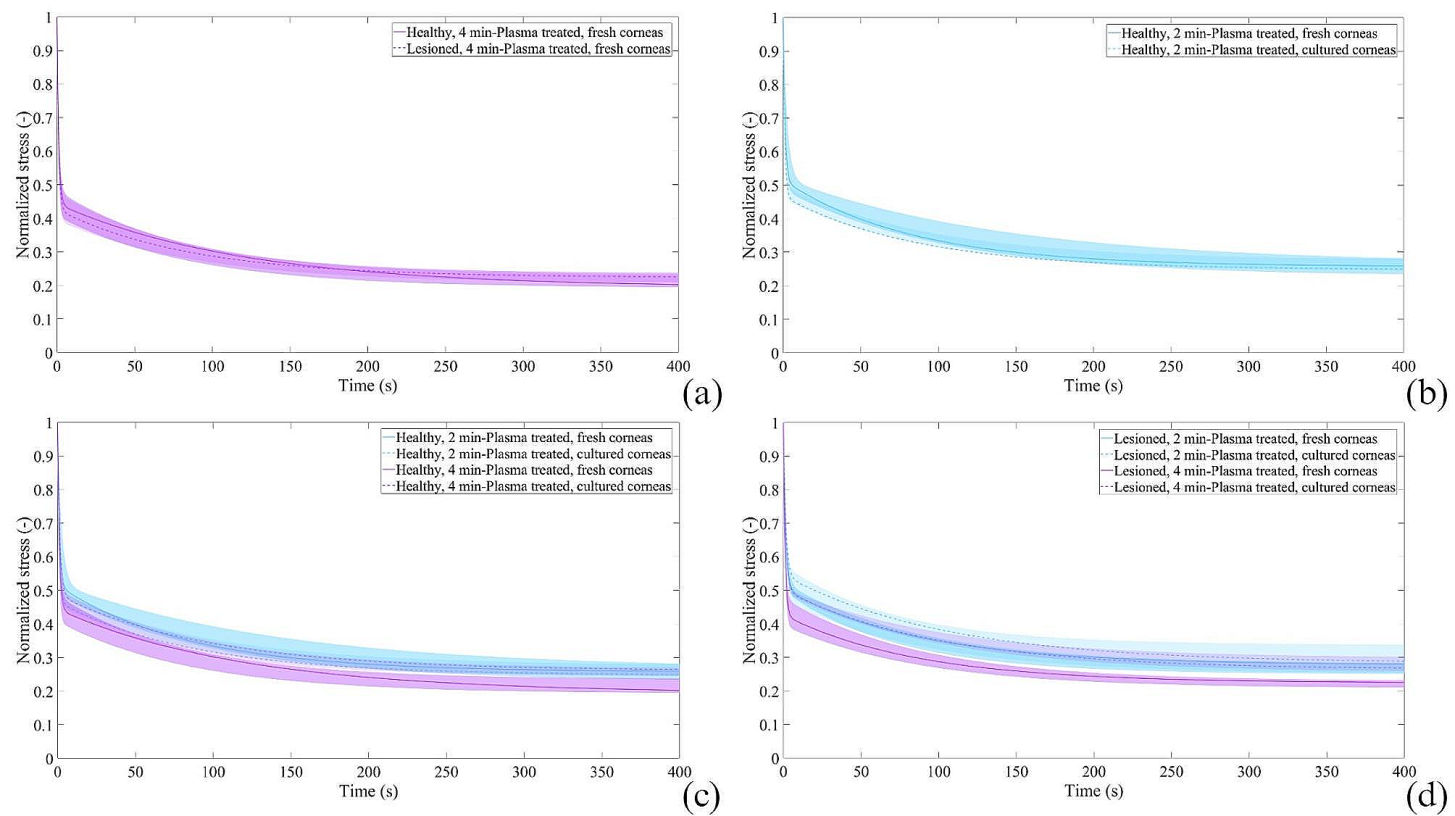
Normalized stress-relaxation curves resulted for plasma-treated corneas in case of (**a**) induction of lesion, (**b**) culture preservation and different exposition times for (**c**) healthy corneas and (**d**) lesioned corneas. Median curves are reported together with 50% probability scatter bands

The stress-relaxation curves show a decrease of normalized stress with time, while a constant strain is applied. This time-dependent behaviour is typical of viscoelastic soft tissues. In all data groups, the stress reaches a plateau in the time range of 400 s. As for the stress-strain curves, the median curves for each population are represented by coloured lines and the associated coloured area represents the range of data dispersion within the group. As concerns the relaxation behaviour, by comparing healthy *versus* lesioned (Fig. 2a) and fresh *versus* cultured (Fig. 2b) plasma-treated corneas, no effect of lesion and culture preservation was found. Moreover, the exposition to plasma treatment for 2 or 4 min induced similar stress relaxation over time (Fig. 2c, d).

**Fig. 3:**
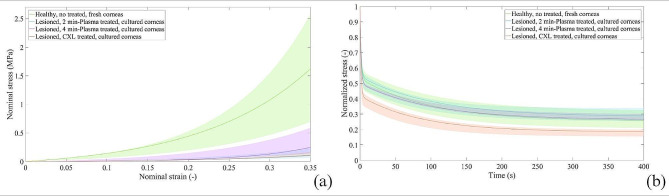
Lesioned plasma-treated in culture corneas results compared to the behaviour of healthy untreated fresh corneas and lesioned CXL-treated in culture corneas. (**a**) Stress-strain curves at equilibrium and (**b**) relative stress-relaxation curves reported in terms of median and associated 50% probability scatter bands

Particular attention was focused on the four populations representing the most typical realistic situation: corneas which were lesioned, cultured and plasma-treated with two different exposition times, namely 2 and 4 min. These results were compared to the behaviour exhibited by healthy untreated fresh corneas and by lesioned CXL-treated corneas (Fig. 3). The comparison was done under the same stress-relaxation protocol. Considering the stress-strain behaviour, the HFN group showed the highest values of stiffness. Comparing the plasma and cross-linking treatment, LCP4 groups showed slightly higher stress values than LCY (Fig. 3a). Considering stress relaxation phenomena, the plasma-treated corneas for both exposition times exhibited a time-dependent behaviour similar to the native tissue (Fig. 3b), contrary to the corneas treated with CXL. The relaxation intensity observed in the plasma-treated (2 and 4 min) groups (F, C, L) are very similar, whereas, comparing the plasma and cross-linking treatment, more intense relaxation phenomena are observed in the LCY group.

The in-culture groups displayed very similar properties to their counterpart fresh ones for 2 min plasma treatment in both healthy and lesioned conditions, while fresh 4 min-plasma treated corneas showed lower values of relaxed stress at the end of the experimental tests, if compared to the in-culture ones (HCP4, LCP4).

### Statistical analysis results

**Fig. 4:**
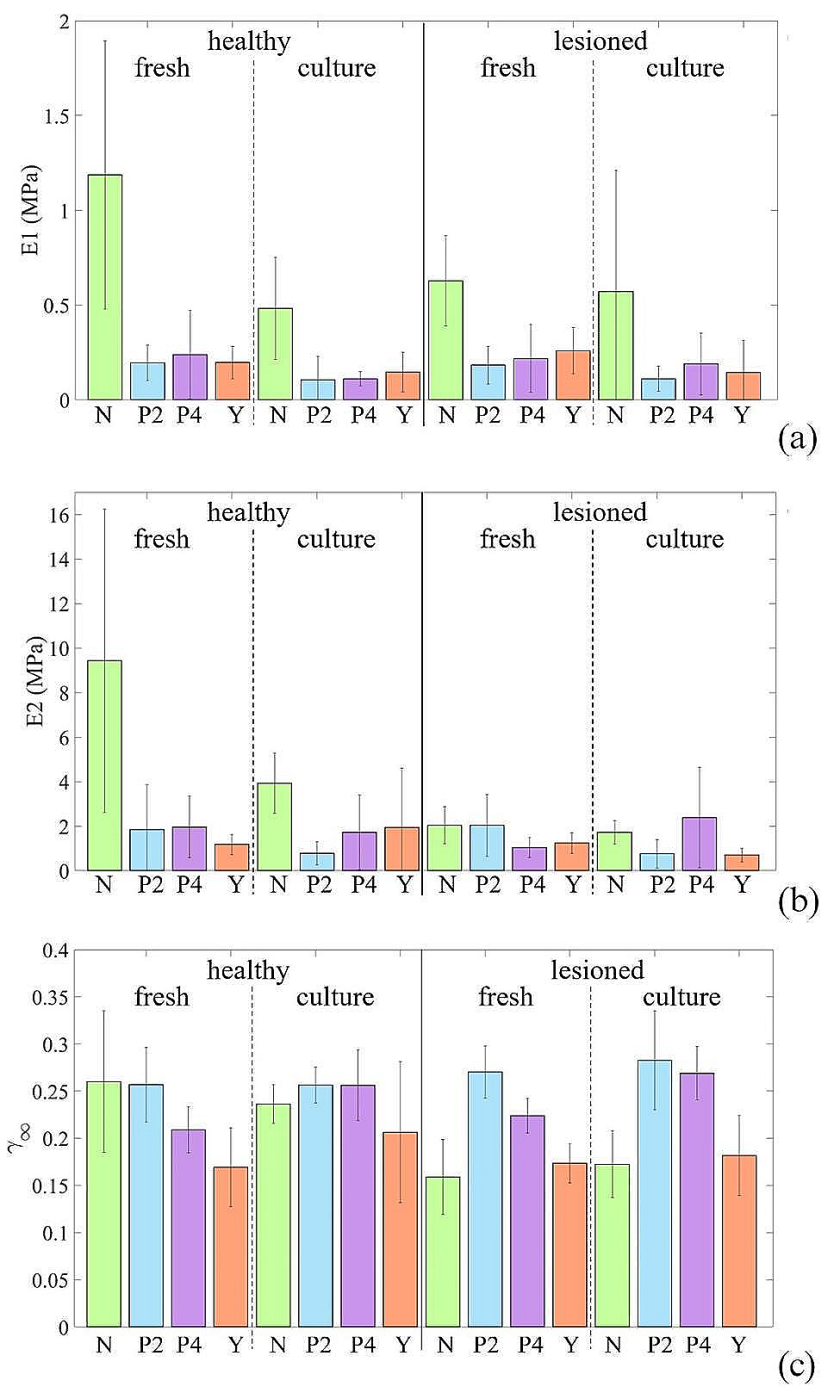
Bar chart (mean ± SD) representative of the tangent modulus (**a**) in the toe region E1 and (**b**) in the linear region E2 for stress-strain curves and (**c**) the parameter γ_∞_ for normalized stress-relaxation curves

From the equilibrium stress-strain curves, the tangent modulus was calculated referring to two different regions, namely the toe and linear regions, for the different groups. The analysis of variance for the toe region parameter revealed that, the healthy population whether consisting of cultured or fresh samples, exhibited a significantly higher values compared to all the other treatment groups, whether considering fresh or cultured and healthy or lesioned samples (Fig. 4a). Notably, among all control groups, HFN had the highest values (1.19 ± 0.71 MPa). Regarding the linear region parameter, the groups’ effect was again significant in the ANOVA analysis primarily driven by HFN which displayed the highest value (9.43 ± 6.82 MPa) and HCN (3.93 ± 1.35 MPa), (Fig. 4b). These values resulted significantly different from all the other groups.

On the other hand, the parameter γ_∞_ at the end of the relaxation phase was obtained from stress-relaxation curves. The γ_∞_ represents the normalized residual stress, i.e., the ability of the material to relax when a constant deformation is applied over time. This parameter highlights the viscosity of the tissue.

By considering γ_∞_ in the different groups, no statistically significant differences were found between the 2 exposure times (2 and 4 min) to the plasma treatment (Fig. 4c). The degree of relaxation in healthy and lesioned plasma-treated corneas for both the exposure times resulted not statistically different compared to the control group (HFN), independently of the preservation method (fresh/in culture). Under the condition of lesion and culture preservation, the γ_∞_ exhibited by the plasma-treated corneas (LCP2 = 0.28 ± 0.01 and LCP4 = 0.27 ± 0.02) resulted significantly higher than the untreated corneas (LCN = 0.17 ± 0.02; *P* = 0.002 and *P* = 0.012 respectively), as reported in Fig. 4c. Moreover, comparing the two corneal treatments considered (LCP2 and LCP4 *versus* LCY), CXL treated groups showed a degree of relaxation significantly higher that the plasma treated (CXL = 0.17 ± 0.01; *P* < 0.001 and *P* = 0.035 respectively).

## Discussion

In the current literature, plasma treatments have been studied in human corneal cells cultures and donated human corneas under microbiological, regenerative and safety aspects [26, 28, 39–41], without investigating the effect on the viscoelasticity of the tissue. In the present study, the results of stress-relaxation tests carried out on porcine corneas first alkali-lesioned and then treated with plasma confirmed the typical non-linear and time-dependent behaviour of the cornea, reported in literature [30–32, 38, 42].

In addition, the current work demonstrates that the exposure of lesioned corneas to the plasma treatment modifies the biomechanical properties of the corneal tissue, if compared to the injured ones. In particular, the plasma treatment restored the relaxation behaviour of the corneal tissue exhibiting values of the equilibrium normalized stress similar to the native tissue (Fig. 4c) [32, 34]. This result is well highlighted by the parameter γ_∞_ that appears to be the same between healthy corneas and those treated with plasma after being damaged.

This viscoelasticity restoration could be the consequence of variations in the chemical components of the corneal stroma after the exposition to the many ROS species that are generated by the plasma flow. In fact, it is well known that ROS are involved in wound healing acting like molecules signals regulators for cell migrations and neoangiogenesis and showing an antimicrobial effect on the tissue [23–25, 27, 28, 41, 39, 43]. 

This result is favourable to the development of a new treatment for melting corneal ulcers, because it could help in preventing corneal perforation by enhancing the stiffness of the tissue. By weighing up plasma and CXL treatments, the obtained results showed that the cross-linking was less effective in terms of bringing the mechanical properties of the tissue back to original normal values. This outcome could lead ophthalmologists to question the choice of CXL for the treatment of corneal infections in favour of plasma.

However, this study has some limitations, such as the time of the culture that was conducted only for 7 days to prevent corneal infections and ensure the vitality of the tissue (more realistic situation). Further investigations are needed to study if a longer conservation after the plasma and CXL treatments affects the mechanical results. Moreover, this is an ex vivo study, so the influence of regenerative processes, such as neoangiogenesis and cell proliferation, that normally take place in vivo situations could not be considered.

To reduce the bias of this study all the present samples and the previous published ones were strictly managed under the same experimental conditions in different days and with different operators; increasing the number of the samples in future research could be helpful to minimize statistical errors.

In order to better understand how the plasma treatment affects the biomechanical properties of the corneal tissue, inflation tests are under investigation. This biomechanical test can better mimic how the tissue responds to elongation in its normal shape and in multiaxial loading conditions and could add other, more realistic information for further clinical applications.

Moreover, to investigate the anatomical effects of plasma in the corneal tissue, histopathology and ultrastructural analysis will be the next steps of our research, in order to evaluate if plasma treatment leads to higher tissue compactness, as already seen in a previous study on CXL effects [44]. Indeed, Perazzi et al. [44] showed by ultrastructural analysis that a higher brightness of a portion of the corneal section examined in that study corresponded to a major damage and a loss of collagen density, while a lower intensity matched a greater stroma compactness. Interestingly, alkali-injured and then CXL treated corneas exhibited brightness values similar to normal tissue ones, suggesting a possible complete recovery after the treatment and demonstrating the ability of riboflavin/UV-A phototherapy to increase stroma collagen.

In human medicine, a histopathologic evaluation of the effect of plasma medicine on organic tissue has been evaluated on the skin, where it has been proved that argon plasma treatment can increase epidermal thickness and dermal collagen density [45]. Considering the biomechanical results of this paper, that lean to a recovery of corneal stiffness after helium-plasma treatment, similar histopathologic results and corneal collagen restoration will be expected too after plasma application.

According to the above, plasma medicine is a new promising regenerative treatment that can improve and faster corneal healing. As a result of future studies and additional experimental tests, the efficacy and security of helium plasma treatment will be further validated before its applications in vivo in animals and human patients with corneal collagenasic ulcers.

## Conclusion

This is the first experimental study that investigates the effects of a helium plasma treatment on the biomechanical properties of ex vivo porcine corneas and it is part of a wider research on the current and the next innovative regenerative medicine techniques. Plasma treatment demonstrated to have an effect on the mechanical properties of the corneal tissue, in terms of increasing its stiffness even after only 2 min of application and recovering the physiological viscoelastic properties of the tissue, suggesting to be a promising possible alternative regenerative treatment to keratoplasty in melting corneal ulcers, by potentially preventing corneal perforation.

## Methods

### Porcine organ-culture preparation procedure

52 porcine eyes were collected from an affiliate slaughterhouse close to the University of Veterinary Medicine in Padua, according to Italian and European law (86/609/EEC). As this is an ex vivo study without any animal sacrifice, no ethical approval and review was necessary. All the samples were considered similar because came from animals of the same age, breed and weight at slaughtering. The eyes were stored immediately after enucleation in a 10% povidone iodine solution and shipped refrigerated at 4 °C to the laboratories for being processed.

The samples were equally and randomly divided into eight experimental populations, considering healthy (H) or lesioned (L) corneas, fresh (F) or culture (C) conditions and plasma treatments with different exposure times, namely 2 (P2) and 4 min (P4).

**Fig. 5:**
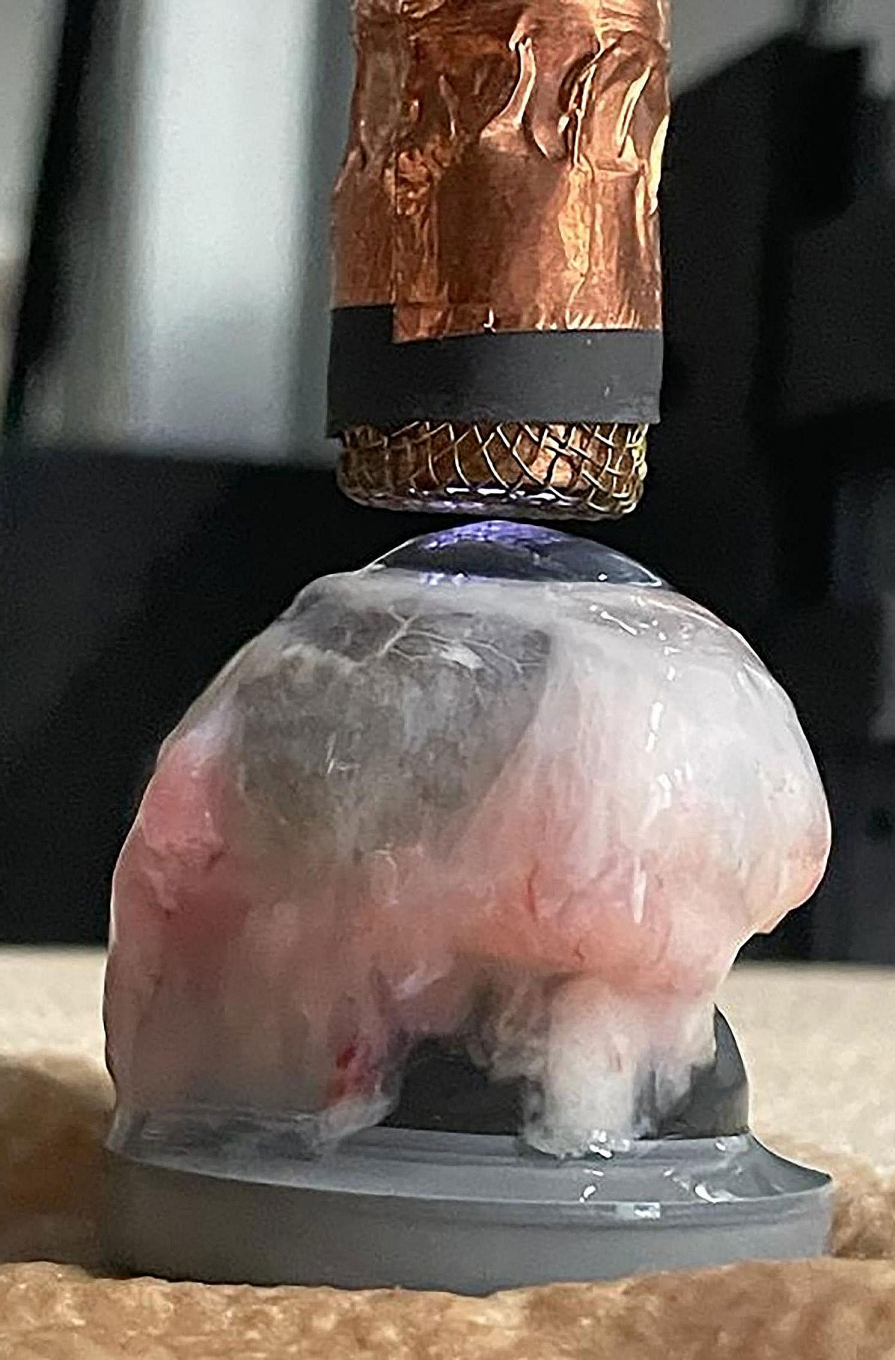
Exposition of the cornea to the plasma treatment

Healthy corneas were tested in their physiological state, whereas lesioned corneas were tested after the exposure of their central portion to a paper filter (0,8 mm) soaked with sodium hydroxide (NaOH) for 1 min to induce an alkali-like experimental lesion, that is chemically comparable to a melting corneal ulcer. The plasma treatment was performed using a plasma source previously described [46] and tested both in vitro [41] and in vivo [39]. The treatment consisted in the close exposure (at a distance equal to 2 mm) of the central portion of the cornea (a circular region of around 1 cm diameter) for 2 or 4 min to a 1.75 L/min helium gas flow passing through a couple of parallel grids driven by a 4.5 MHz radio frequency sinusoidal voltage difference of approximately 1 kV peak-to-peak (Fig. 5). The plasma produced between the two grids is enriched in ROS and RNS, due to the air traces which mix with the helium flow. The sample is then exposed to the afterglow. The plasma treatment takes place rinsing with sterile PBS (Dulbecco’s PBS; PAA Laboratories) the surface of the cornea every minute to prevent dryness. Fresh corneas were tested within 6 h from harvest and were maintained soaked in physiologic saline solution at room temperature prior to tests, culture populations after 7 days of preservation in a specific medium (CARRY-C®, Alchimia, Padua) to maintain the native biological characteristics of the tissue. Before being stored as described above, to reduce the risk of tissue contamination, the corneas were isolated from the entire bulbs, maintaining approximately 4 mm of peri-limbal sclera. Then, each sample were suspended into a sterile bottle filled with 25 ml of preservative medium with sutured thread passing through the adjacent sclera and incubated at 37 °C with 5% CO_2_. A daily check was performed during the culture period: if the colour of the medium moved from red to yellow, indicating contamination of the tissue, the sample was eliminated from the study.

Moreover, the results on the biomechanical behaviour of additional 42 porcine corneas previously described and tested [38] were added to the analysis, thus including also non-treated (N) and CXL-treated (Y) populations (Vetuvir®, vision Engineering Italy srl, Rome, Italy). As comprehensively described in [38], N corneas didn’t receive any treatment, while the central portion of the Y corneas was wet for 30 min with an isoosmolar 0.1% riboflavin solution (Peschke Traid, Huenenberg, Switzerland) administered in a circular plastic well held in contact with the surface of the tissue to promote the imbibition of the stroma. Then, with a commercially available equipment, the corneas were irradiated at a distance of 10 cm for 3 min with 30mW/cm2 UV-A of 365 nm wavelength and a total energy of 5,4 J/cm2. As for the P populations, a 1 min PBS flush was set at the end of the treatment.

Therefore, the corneas were considered in healthy status, non-treated or after the plasma or CXL treatment in fresh (HFN, HFP2, HFP4, HFY) and culture (HCN, HCP2, HCP4, HCY) conditions and similarly for the lesioned status (LFN, LFP2, LFP4, LFY, LCN, LCP2, LCP4, LCY).

### Mechanical tests

**Fig. 6:**
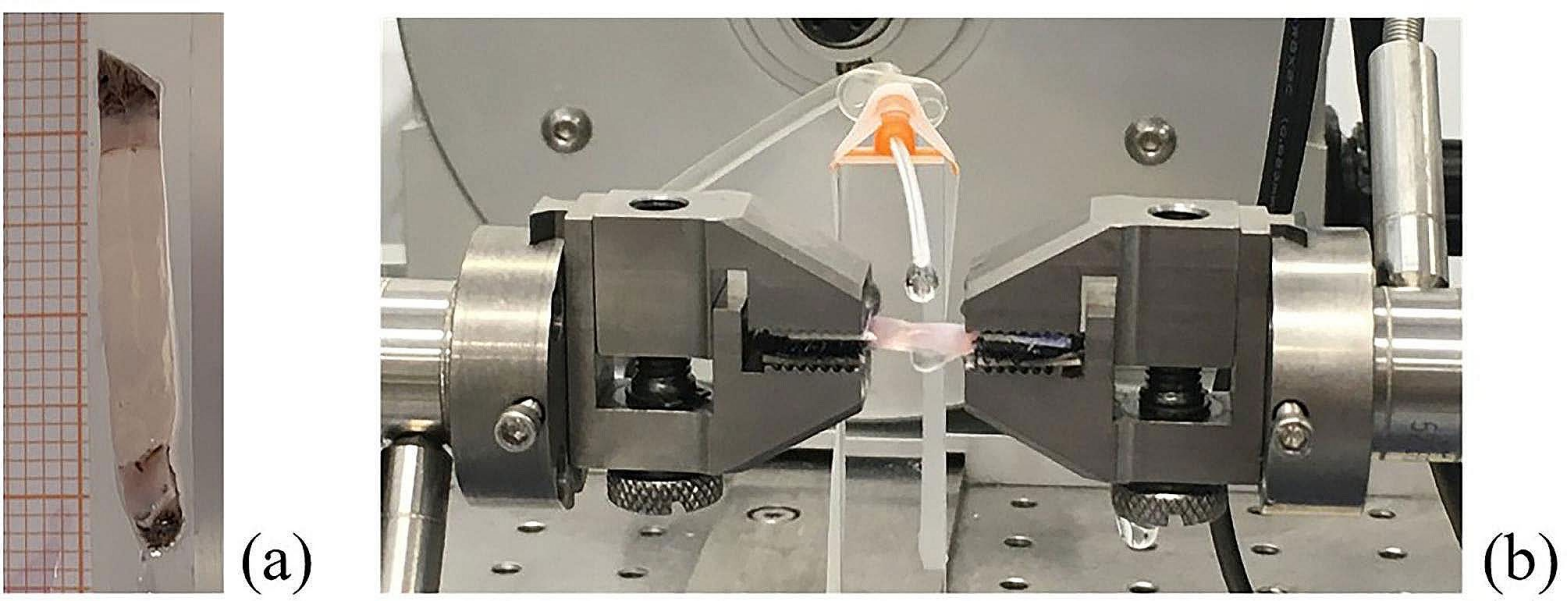
(**a**) Geometry of corneal specimen for (**b**) mechanical tensile test

From each cornea, a single corneal sample was obtained for mechanical testing. Each sample was trimmed using a specific cutting die for a rectangular shape (width 4 mm, length 10 mm). The corneal samples were cut along the medial/lateral direction, leaving a small amount of sclera for gripping (Fig. 6a).

The mechanical tester used was a Bose ElectroForce^®^ Planar Biaxial Test Bench (TA Instruments, New Castle, USA), equipped with a load cell of 22 N. Mechanical tests were carried out following a protocol already described [38]. Before testing, sample thickness was measured using a digital calliper at different positions, and then each end of the samples was interposed between two patches of balsa wood, to which the male sides of the Velcro were glued. A medical-grade cyanoacrylate glue (Glue Loctite 4013 Prism) was used to fix the ends of the samples on Velcro surfaces. Finally, the samples were positioned within the grips of the mechanical tester, aligned and a closure pressure was adjusted to avoid slippage. During testing, samples were continuously hydrated by dropping the solution on the sample surface (as showed in Fig. 6b).

All the samples were subjected to a first preconditioning phase through the application of 10 loading-unloading cycles up to 8% strain, at a strain rate of 1%/s. Then, the stress-relaxation protocol included four consecutive steps of almost instantaneous strain (strain rate of 800%/s), with each step of 8% strain, which was held constant for a time interval of 400s .

### Post-processing technique

**Fig. 7:**
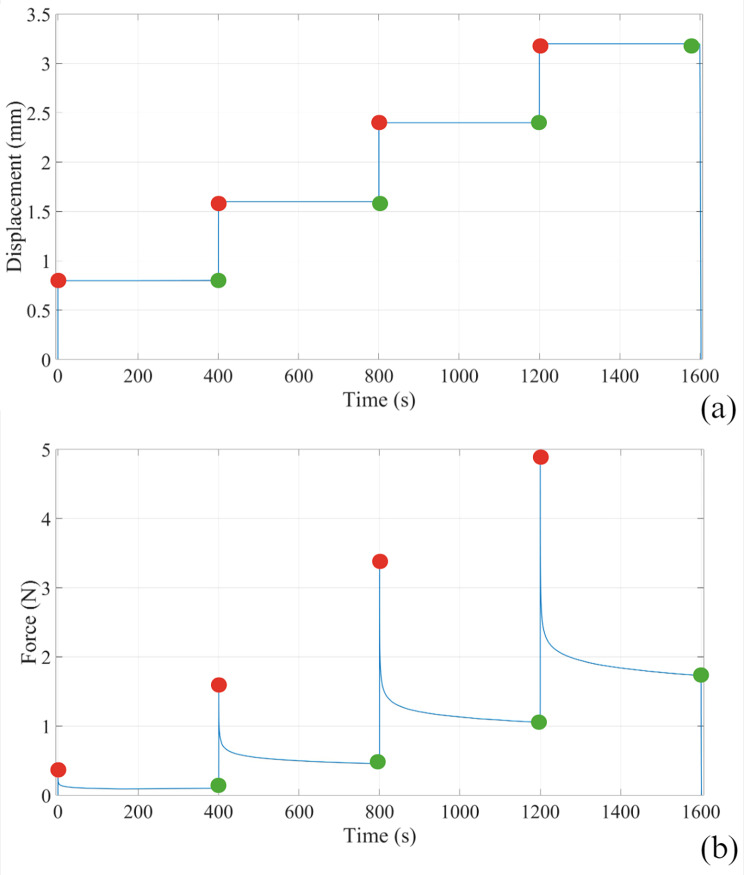
Depending on the (**a**) strain history, typical force-time data measured for the stress-relaxation test. In red the peak loads corresponding to the quasi-instantaneous application of the displacement level while in green the equilibrium loads after 400 s

Experimental force-time data, depending on the assumed strain history, were obtained from each test (Fig. 7). Force was then converted to stress, as the ratio between force and the initial cross-sectional area. The equilibrium stress-strain curves were obtained considering the end of relaxation phases and the corresponding applied strain, leading to the mechanical response when all the time-dependent phenomena completely occur. The corneal viscoelastic response was analysed considering stress *versus* time curves. Stress relaxation data were processed by computing the normalized stress as the ratio between the current stress and peak stress of each rest phase [38] and then, the normalized stress-time curves were separately fitted by the following exponential formulations:

1 $${\rm{\sigma norm}}\,\left( {\rm{t}} \right)\,{\rm{ = }}\,{\rm{1}}\,{\rm{ - }}\,{\rm{\gamma 1}}\,{\rm{ - }}\,{\rm{\gamma 2}}\,{\rm{ + }}\,{\rm{\gamma 1}}\,{\rm{exp}}\left( {{\rm{ - }}\,{\rm{t/\tau 1}}} \right)\,{\rm{ + }}\,{\rm{\gamma 2}}\,{\rm{exp}}\left( {{\rm{ - }}\,{\rm{t/\tau 2}}} \right)$$

where parameters γ1,γ2 represent the relative stiffness and τ1,τ2 the time constants of relaxation. From the equilibrium stress-strain curves, two tangent moduli were calculated as the slope of the curve in the toe region (E1) and in the linear region (E2), while for the normalized stress-time curves the attention was focused on the equilibrium region by considering the equilibrium normalized stress γ∞, computed as:

2 $${{\rm{\gamma }}_\infty }{\rm{ = 1}}\,{\rm{ - }}\,{\rm{\gamma 1}}\,{\rm{ - }}\,{\rm{\gamma 2}}$$

### Statistical analysis

For the statistical analysis, all of the corneal samples described in the methods section were included. Totally, 94 samples from 16 levels were considered from the combination of 3 factors: healthy *versus* lesioned (population factor: 2 levels), culture *versus* fresh (condition factor: 2 levels), N *versus* P2 *versus* P4 *versus* Y (treatment factor: 4 levels). The regression between stress-strain or stress relaxation and time were analysed using a non-linear model. Two parameters of the stress-strain curves (E1 toe region and E2 linear region) and one of the relaxation curves (γ_∞_, plateau of the curve) were calculated. The parameters estimated for the 94 individual curves were subjected to ANOVA, considering the fixed effect of the 16 combined levels. Post-hoc pairwise comparison among levels was performed using the Bonferroni correction. A *p*-value of less than 0.05 was considered a significant change. All the analyses were performed using SAS software (SAS Institute Inc., Cary, NC, USA, 2002–2012).

## Data Availability

The datasets analysed during the current study are available from the corresponding author on reasonable request.
